# M2 macrophages secrete CXCL13 to promote renal cell carcinoma migration, invasion, and EMT

**DOI:** 10.1186/s12935-021-02381-1

**Published:** 2021-12-18

**Authors:** Yingwei Xie, Zhiliang Chen, Qiyu Zhong, Zaosong Zheng, Yuqing Chen, Wentai Shangguan, Yishan Zhang, Jingying Yang, Dingjun Zhu, Wenlian Xie

**Affiliations:** 1 Department of Urology, Sun Yat-sen Memorial Hospital, Sun Yat-sen University, Guangzhou, 510120 China; 2 Guangdong Provincial Key Laboratory of Malignant Tumor Epigenetics and Gene Regulation, Sun Yat-sen Memorial Hospital, Sun Yat-sen University, Guangzhou, 510120 China; 3 Guangdong Provincial Clinical Research Center for Urological Diseases, Sun Yat-sen Memorial Hospital, Sun Yat-sen University, Guangzhou, 510120 China; 4 Department of Pathology, Sun Yat-sen Memorial Hospital, Sun Yat-sen University, Guangzhou, 510120 China; 5 Department of Urology, Nanfang Hospital, Southern Medical University, Guangzhou, 510515 China

**Keywords:** Tumor microenvironment, Tumor-associated macrophages, CXCL13, ccRCC, Progression

## Abstract

**Objective:**

M2 macrophages are associated with a poor prognosis in a variety of malignancies. There are, however, few relevant investigations in clear cell renal cell carcinoma (ccRCC).

**Methods:**

The expression of M2 macrophages in ccRCC tissues was first discovered using immunohistochemistry in this study. Then, M2 macrophages were created in vitro to see how they affected the proliferation, migration, invasion, and EMT of ccRCC cells. Using qPCR and prognostic analysis identifies important chemokine. Antibody neutralization tests confirmed the chemokine’s involvement and function. Pathway inhibitors confirmed the main pathway of M2 macrophages in ccRCC. Finally, qPCR and IHC were used to confirm the expression of chemokine receptors in ccRCC tissues.

**Results:**

The presence of M2 macrophages was linked to a poor outcome in ccRCC. M2 macrophages enhanced the proliferation, migration, invasion, and EMT of ccRCC lines in vitro. CXCL13 was identified as the main chemokine by prognostic analysis and qPCR tests. CXCL13 neutralizing antibodies can inhibit the stimulation of M2 macrophages in ccRCC lines’ proliferation, migration, invasion, and EMT. M2 macrophages and CXCL13 may activate the Akt pathway in ccRCC lines, and Akt inhibitors decrease ccRCC lines proliferation, migration, invasion, and EMT. CXCR5 expression is a poor prognostic factor for renal cell carcinoma, according to qPCR and immunohistochemistry. In vivo experiments further proved that CXCL13 secreted by M2 macrophages can promote tumor proliferation.

**Conclusions:**

M2 macrophages in the immunological milieu secrete CXCL13, which promotes ccRCC proliferation, migration, invasion, and EMT. Our findings contribute to a better understanding of the function of the tumor microenvironment in the incidence and progression of ccRCC, and they may point to novel therapeutic targets for ccRCC.

**Supplementary Information:**

The online version contains supplementary material available at 10.1186/s12935-021-02381-1.

## Background

Renal cell carcinoma (RCC) accounts for around 2–3% of all malignant tumors worldwide, and its prevalence is rising. Metastatic renal cell carcinoma accounts for 25–30% of all RCC and has an exceedingly poor prognosis [1]. In 2020, there will be roughly 430,000 new instances of RCC found worldwide, with 179,000 fatalities as a result of RCC [2]. Clear cell renal cell carcinoma (ccRCC) is the most prevalent form of RCC, accounting for about 70–80% of all cases [3]. As a result, determining the mechanism and molecular indicators of ccRCC metastasis is critical to improving the prognosis of ccRCC patients.

RCC is an immunogenic tumor, and immunotherapy has long been an essential part of RCC treatment [4]. The tumor microenvironment (TME), which is composed of mesenchymal cells and immune cells that have been recruited, has immunosuppressive and tumor-promoting effects [5]. Tumor-associated macrophages (TAM) are among the most numerous cells in TME. M2 macrophages, the major component, have been recognized as a poor prognostic factor for a variety of malignancies, including RCC [5–7]. However, the mechanism through which TAM contributes to tumor development is unknown. M2 macrophages in TAM have been demonstrated in several studies to have a role in tumor growth, invasion, and metastasis through chemokines [8, 9]. However, research on TAM’s function in ccRCC is currently limited.

Chemokine ligand 13 (CXCL13) belongs to the CXC chemokine family, and chemokine receptor 5 is its particular receptor (CXCR5). In a variety of malignant tumors, CXCL13 interacts with its receptor CXCR5 to promote carcinogenesis, development, and metastasis [10]. CXCL13 has previously been shown to be a poor prognostic factor for ccRCC and to enhance the proliferation of ccRCC cells [11]. We also discovered that M2 macrophages may release a lot of CXCL13, which promotes the proliferation, invasion, and metastasis of ccRCC.

## Methods

### Patient tissue sample

This study was approved by the Sun Yat-sen Memorial Hospital Ethics Committee. From September 2011 to March 2021, our hospital (SYSMH) collected 190 ccRCC tissues and their matching adjacent tissues. The 190 ccRCC patients included 111 men and 79 women of age 21–89 years (average age: 53.2 years). Written informed consent was obtained from all participants. The patient inclusion criteria included clear cell renal cell carcinoma. Exclusion criteria included patients who did not receive any anti-tumor therapy before surgical resection. The pathologist of our hospital confirmed the ccRCC tissues and the adjacent non-tumor tissues.

### Database

The database information used in this study is TCGA (https://portal.gdc.cancer.gov/). Since the data was sourced from an open public database, there was no need to obtain research approval from the ethics committee.

### Reagents and antibodies

Recombinant human CXCL13 (300-47-50) was purchased from PeproTech (New Jersey, USA). PMA was obtained from Sigma (Darmstadt, Germany). The following antibodies are used for western blotting (WB), neutralization assay, flow cytometry, enzyme-linked immunosorbent assay (ELISA), and immunohistochemistry (IHC): CD11b and CD206 (BD Biosciences, San Diego, USA); CXCL13 ELISA (ab179881), rabbit anti-CXCL13 (Ab272874), rabbit anti-CXCR5 (ab203212), mouse anti-GAPDH (ab8245), rabbit anti-AKT (11E7; CST); rabbit anti-p-AKT (Ser473; 4060, CST), rabbit anti-E-cadherin (3195; CST), rabbit anti-N-cadherin (13116; CST), rabbit anti-vimentin (5741; CST), and rabbit anti-Ki67 (ZA-0502, ZS, China). Akt inhibitor A3149 was purchased from APExBIO (Houston, USA).

### Cell culture

The human ccRCC cell lines Caki-1, ACHN, and human THP-1 were obtained from the American Type Culture Collection (ATCC; Manassas, VA, USA). All cell lines were cultured in RPMI 1640 medium (Gibco), supplemented with 10% fetal bovine serum (FBS; Hyclone Technologies) and 1% penicillin and streptomycin. All cell lines are maintained in a humid environment at 37 °C and under a 5% CO_2_ atmosphere.

### THP-1 cells induced differentiation

The THP-1 cells were collected and inoculated into 6-well plates and then induced with PMA (100 ng/mL). M0 macrophages were obtained after culturing for 48 h. The Caki-1 and ACHN cells were seeded in the upper cavity of a Boyden chamber (0.4-µm pore size; Corning, USA) and co-cultured with M0 macrophages for 4 days to obtain M2 macrophages, after which flow cytometry was performed to determine whether the induction was successful.

### Conditioned medium (CM)

After the macrophages are induced to differentiate, and then the macrophages were washed with PBS. Then, a fresh medium was added to the cells, followed by incubation for another 24 h. The medium supernatant was harvested and filtered, after which the complete medium was added at a ratio of 30% to produce the corresponding conditioned medium (M0-CM and M2-CM).

### Co-cultivation program

The ccRCC cells and THP-1-derived macrophages were co-cultured in a Boyden chamber. M0 and M2 macrophages were seeded in the upper chamber, and the Caki-1 and ACHN cells were seeded in the lower chamber. Next, recombinant human CXCL13, CXCL13-neutralizing antibody, or AKT inhibitor A3149 were added to the lower chamber and incubated for 12 h for co-cultivation. The RCC cells in the lower chamber were finally collected for PCR or WB.

### RNA extraction and quantitative PCR (RT-qPCR)

TRIzol (Thermo Fisher Scientific) was used to extract the total RNA from cultured cells or tissues. With reference to the manufacturer’s protocol, total RNA was reverse-transcribed into cDNA using the Prime Kit (Takara Biotechnology). The ABI7500 real-time PCR system (Thermo Fisher Scientific) was used to perform PCR with SYBR premixed real-time fluorescent quantitative PCR reagents (Taojiu Biotechnology). GAPDH was used as an internal control. The comparative 2^−ΔΔCq^ method was used for relative quantification. The primer sequences of the analyzed genes are shown in Additional file 1.

### Western blotting analysis

The total protein was separated from the cells with RIPA lysis buffer, separated by polyacrylamide gel, and then transferred onto the PVDF membrane. After blocking with 5% BSA solution, the membrane was incubated with primary antibodies (including E-cadherin, N-cadherin, Vimentin, AKT, and p-AKT) overnight. This membrane was then incubated with HRP-conjugated anti-rabbit antibody or anti-mouse antibody for 1 h at room temperature. The super signal WestFemto Maximum Sensitive Substrate (Thermo Fisher Science) was used to detect protein bands and the images were captured. The original western blots with markers are shown in Additional file 2.

### MTS determination

Cell proliferation was measured by the MTS assay. Cells (1 × 10^3^; 100 µL per well) were seeded into 96-well plates. Different groups of cells were added with different (fresh) conditioned media (PBS, M0-CM, and M2-CM) and the corresponding additives (recombinant human CXCL13, neutralizing antibody of CXCL13, or AKT inhibitor A3149) daily. Next, 20% MTS solution was added to each well, followed by incubation at 37 °C in the dark for 2 h. The optical density of the cells was then measured at 492 nm. The measurements were performed at 0, 24, 48, 72, 96, and 120 h to generate growth curves.

### Wound healing assay

The cells were seeded in 12-well plates and cultured to produce a confluent monolayer. By using a 200-µL pipetting tip, the wound area was scratched and then collected tissues were washed thrice in PBS to remove debris. The washed cells were placed in a 12-well Boyden chamber and then inoculated with M0 and M2 macrophages in the upper chamber and FBS-free medium and additives (recombinant human CXCL13, neutralizing antibody to CXCL13, or AKT inhibitor) in the lower chamber. The wound closure was observed under an inverted microscope and the images were captured during 0–12 h.

### Migration and intrusion detection

In the migration test, 1 × 10^5^ cells were suspended in 200-µL of serum-free medium per well and seeded in the upper chamber of a 24-well Boyden chamber. In the invasion test, 1 × 10^5^ cells suspended in 200 µL of serum-free medium were inoculated into the upper chamber of a Matrigel-coated cell. At the beginning of the cell migration assay, 1 × 10^4^ M0 and M2 macrophages were seeded in the lower compartment. Next, recombinant CXCL13, anti-CXCL13 neutralizing antibody, and Akt inhibitor were added to the lower compartment. After 48 or 72 h of incubation, the membrane was fixed with 4% paraformaldehyde and then stained with crystal violet solution. At least 5 randomly selected areas were counted at a magnification of 100×.

### ELISA

According to the manufacturer’s instructions, the secretion of human CXCL13 in the supernatant of M0 and M2 macrophages was detected. After color development, the absorbance at 450 nm was measured on a microplate reader.

### Flow cytometer

According to the manufacturer’s instructions, the digested cells were washed with PBS and incubated with flow cytometry antibodies CD11b and CD206 for 20 min in the dark, followed by washing with PBS and resuspension on the machine. Finally, the CellQuest software version 7.5.3 (FACS Vantage-SE, BD Immunocytometry Systems, San Diego, CA) was employed to analyze the cells by multicolor flow cytometry. The flow cytometry gating strategies are depicted in Additional file 3.

### Immunochemistry

The ccRCC and adjacent tissues were fixed with formalin, and the paraffin-embedded tumor tissues and the adjacent sections were stained with anti-CD206 antibody, anti-Ki67 antibody, anti-E-cadherin and anti-CXCR5 antibody, respectively. CD206 was used as a marker for M2 macrophages. The stained cells were counted in each field of view, and each slice had a field of view of at least 400 times. The average number of positive cells from the 5 highest field measurements was used for subsequent data analysis. The expression of the target protein was evaluated by the ratio and intensity of positive cells.

### Xenotransplantation experiment

All animal experiments were performed following the protocol approved by the Animal Care and Use Institutional Committee of Sun Yat-sen Memorial Hospital, Sun Yat-sen University (Guangzhou, China). In order to study the effect of CXCL13 on tumor growth, 5-week-old female BALB/c nude mice were randomly divided into negative and CXCL13 groups (n = 5 in both groups). Approximately 5.0 × 10^6^ Caki-1 cells were injected subcutaneously into the upper back of nude mice. When the xenograft was palpable (approximately 0.5-cm in diameter), 0.1 mg/kg of PBS, CXCL13 or CXCL13 + A3149 (A3149: 30 mg/kg) was injected weekly for 4 consecutive weeks [9]. The following formula was used to calculate the tumor volume (V) every 3 days: V = (W^2 ^× L)/2. Finally, mice were sacrificed by placing them in a euthanasia chamber introduced with 100% CO_2_ gas at a flow rate of 25% at a chamber volume per minute for 4 min.

### Statistical analysis

All statistical calculations were performed using the SPSS 20.0 (IBM Corp.) and R software. Quantitative data and categorical data were analyzed by Student’s *t*-test and Fisher’s exact test, respectively. One-way analysis of variance and Tukey’s post-test were performed for statistical analysis on more than 2 groups. The cumulative survival rate was calculated by Kaplan–Meier analysis and log-rank test. *P *< 0.05 was considered to indicate a statically significant difference.

## Results

### Increased M2 macrophage infiltration is linked to a poor outcome in patients with ccRCC

**Fig. 1:**
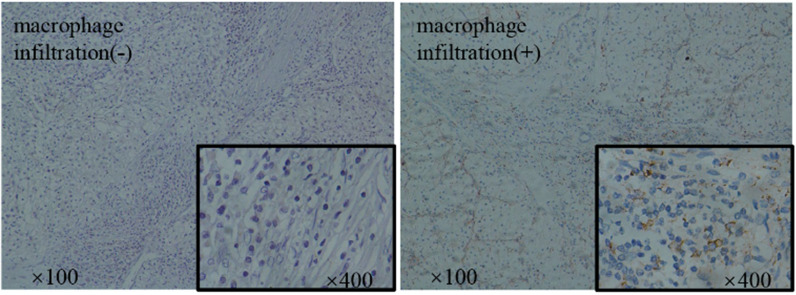
M2 macrophage infiltration is increased in ccRCC. Immunohistochemical staining of CD206 protein (M2 macrophage marker) in ccRCC tissue

To see if M2 macrophage infiltration is linked to ccRCC tumorigenesis and progression, we utilized CD206 protein as an M2 macrophage marker and assessed M2 macrophage infiltration in 55 ccRCC samples using IHC (Fig. 1). The degree of M2 macrophage infiltration was positively correlated with the pathological grade and T stage of ccRCC patients, according to the findings (Table 1). These findings suggest that M2 macrophages play a crucial role in ccRCC tumorigenesis and development.

**Table 1:** Associations of M2 macrophage infiltration with clinicopathological ccRCC features

Characteristics	Number	CD206		*P*-value
		Positive	Negative	
Gender				
Male	30	8	22	0.456
Female	25	9	16	
Age				
≤ 60	39	10	29	0.187
> 60	16	7	9	
Grade				
G1–2	35	6	29	0.003*
G3–4	20	11	9	
T stage				
T1–2	45	11	34	0.028*
T3–4	10	6	4	

*Results were considered statistically significant at *P* < 0.05

### M2 macrophages generated from THP-1 induce EMT in ccRCC cells

**Fig. 2:**
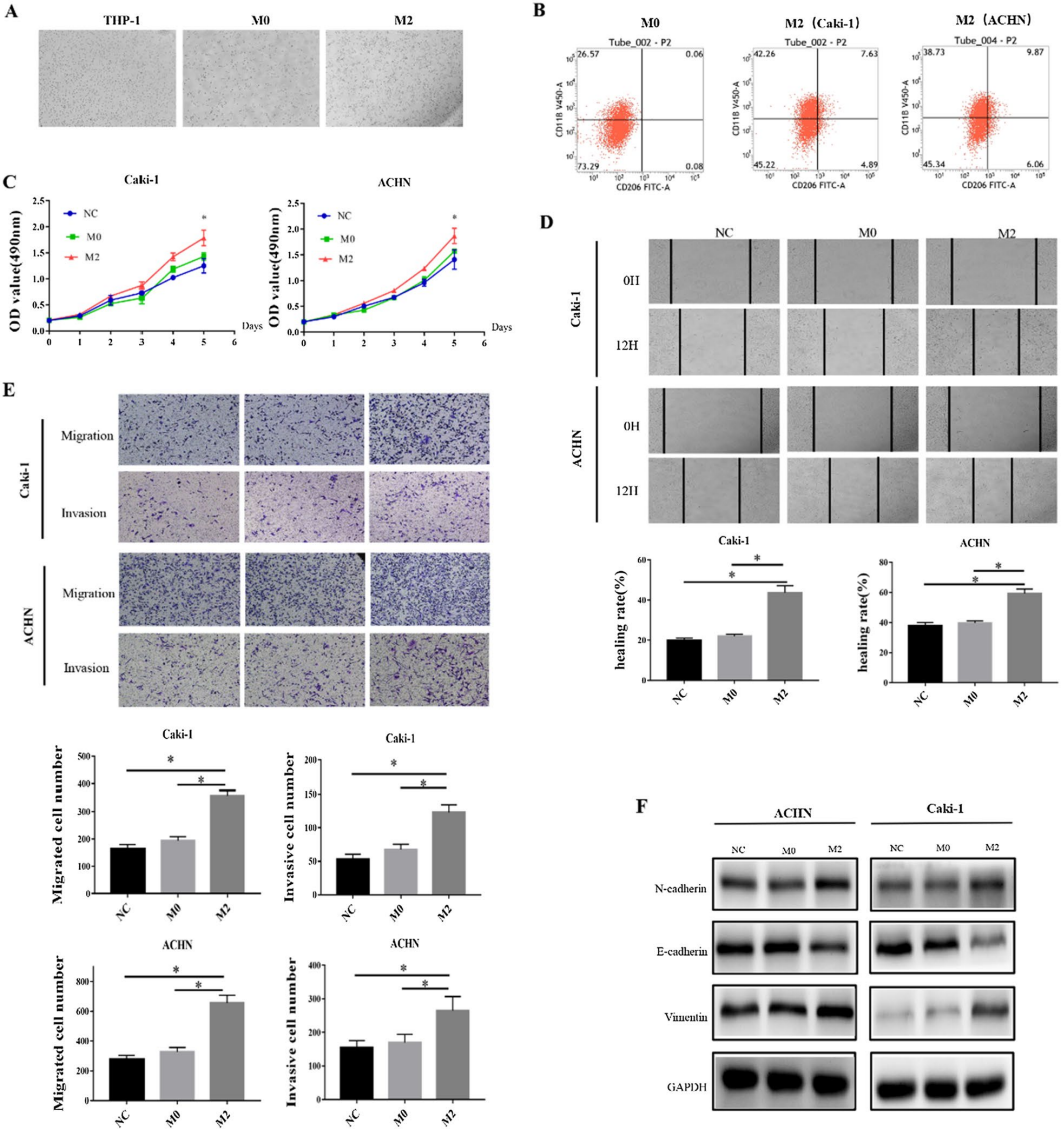
THP-1-derived M2 macrophages promote EMT in ccRCC cells. **A** Cell morphology of THP-1, M0 and M2 cells. **B** Detect the expression of CD11b and CD206 in M0 and M2 macrophages by flow cytometry. **C** MTS detects the effect of M2 macrophages on the proliferation of ACHN and Caki-1. **D** Scratch healing test to detect the effect of M2 macrophages on the migration of ACHN and Caki-1. **E** Transwell chamber experiment to detect the influence of M2 macrophages on the migration and invasion of ACHN and Caki-1. **F** WB experiment to detect the effect of M2 macrophages on ACHN and Caki-1 EMT. (*P < 0.05)

To better understand the function of M2 macrophages in ccRCC. THP-1 was initially converted into M0 macrophages using PMA, and then co-cultured with ccRCC cell lines Caki-1 and ACHN to produce M2 macrophages, which were detected by flow cytometry (Fig. 2A, B), and CD206 positive rates increased substantially, demonstrating that macrophages and ccRCC cell lines may differentiate into M2 macrophages when co-cultured. Through M2 macrophage conditioned medium and co-culture, we discovered that M2 macrophage can enhance Caki-1 and ACHN proliferation, migration, and invasion (Fig. 2C–E).

We investigated the expression of EMT markers in ccRCC since M2 macrophages can enhance the migration and invasion of ccRCC cell lines. Co-culture with M2 macrophages resulted in a substantial reduction in E-cadherin expression in Caki-1 and ACHN cells, whereas N-cadherin and vimentin expression increased at the same time (Fig. 2F). In conclusion, these findings suggest that M2 macrophages may enhance ccRCC cell proliferation, migration, and invasion via EMT.

### Differential expression of chemokines in macrophages and RCC tissues

**Fig. 3:**
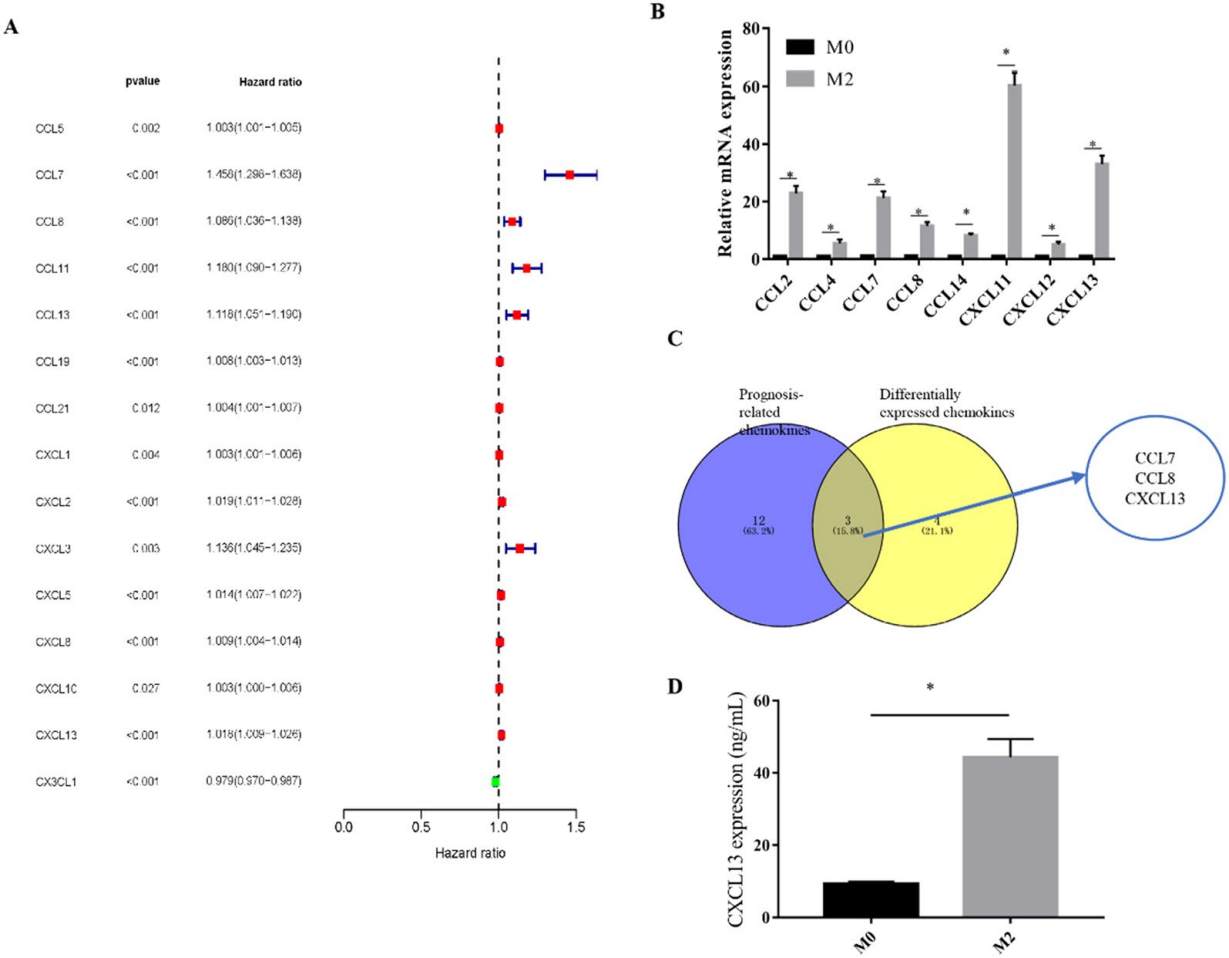
CXCL13 is a key chemokine secreted by M2 macrophages. **A** Univariate prognostic analysis of chemokines on the prognosis of ccRCC. **B** M2 macrophages are highly expressed chemokines compared to M0 macrophages. **C** Venn diagram takes the intersection of diagram A and diagram B. **D** ELISA to detect the expression of CXCL13 in the supernatant of M2 macrophages (*P < 0.05)

Many chemokines are known to be secreted by M2 macrophages to modulate their function in tumor spreading. As a result, we first assessed the effect of different chemokines on the prognosis of ccRCC. The TCGA database was used to evaluate the expression of different chemokines in ccRCC, and the results are given in Additional file 4. Then, using univariate prognostic analysis, we investigated the effect of different chemokines on the prognosis of ccRCC. The findings revealed that 15 chemokines were strongly associated with the prognosis of ccRCC (*P *< 0.05) (Fig. 3A). Using qPCR, we found a variation in Chemokine expression between M2 macrophages and M0 macrophages. The findings revealed that the expression of 7 chemokines rose substantially (Differential expression > 4 times) (Fig. 3B). We use the Venn diagram to select the intersection of Figure A and Figure B, and finally determine the three key chemokines CCL7, CCL8 and CXCL13 (Fig. 3C). We selected CXCL13 with the highest expression difference among the three key chemokines for ELISA experiments, and the results confirmed that M2 macrophages secreted a large amount of CXCL13 (Fig. 3D).

### CXCL13 is an important cytokine for M2 macrophages in promoting EMT in ccRCC

**Fig. 4:**
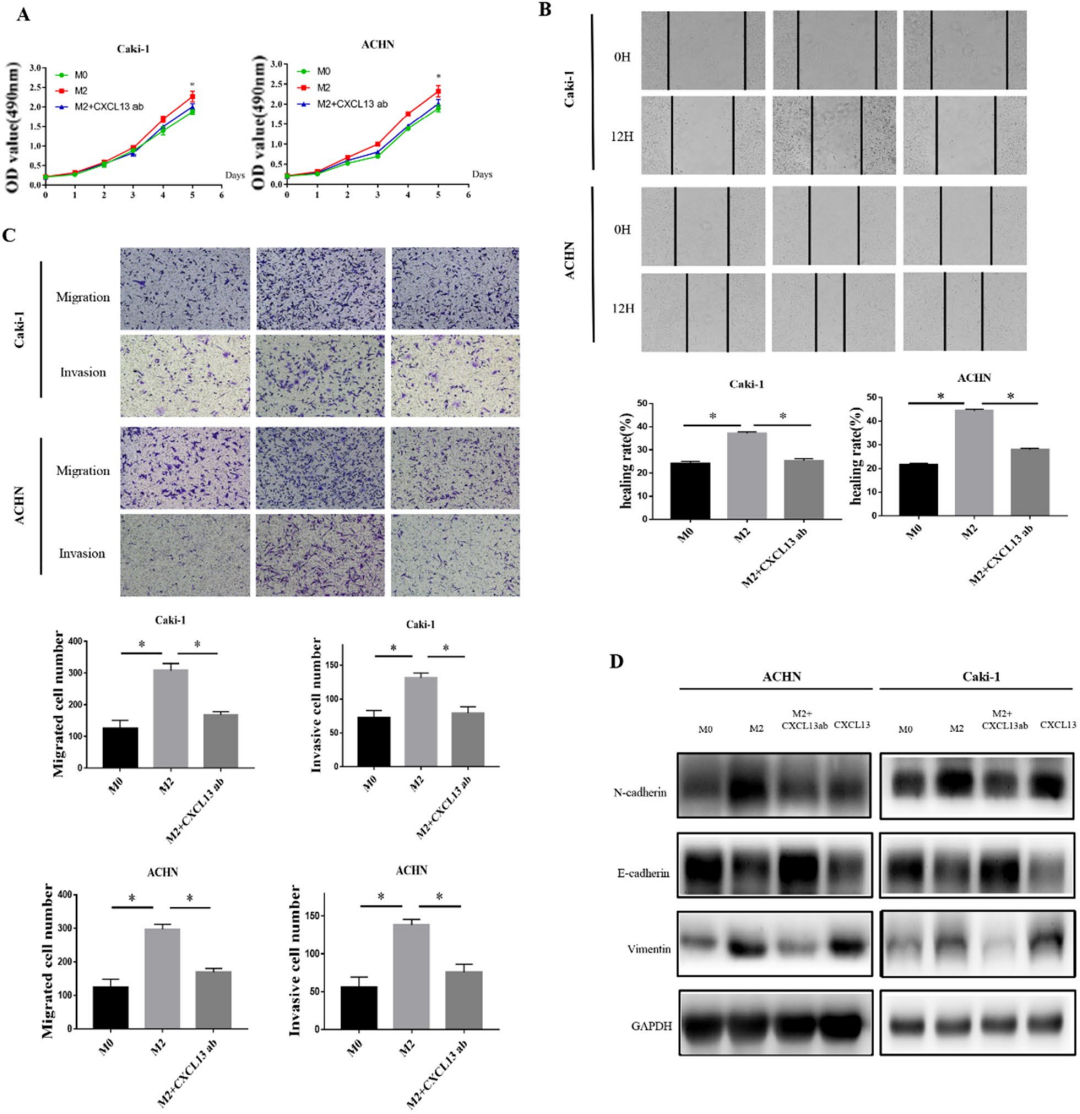
M2 macrophages secrete CXCL13 to promote EMT in ccRCC cells. **A** In the presence or absence of anti-CXCL13 antibodies, MTS detects the effect of M2 macrophages on the proliferation of ACHN and Caki-1. **B** In the presence or absence of anti-CXCL13 antibodies, the scratch healing test detects the effect of M2 macrophages on the migration of ACHN and Caki-1. **C** In the presence or absence of anti-CXCL13 antibodies, the Transwell chamber test detects the effect of M2 macrophages on the migration and invasion of ACHN and Caki-1. **D** In the presence or absence of anti-CXCL13 antibodies or CXCL13, western blotting revealed the effect of M2 macrophages and CXCL13 on ACHN and Caki-1EMT (*P < 0.05)

Based on the data above, we tried to investigate if CXCL13 is required for M2 macrophages to induce ccRCC EMT. As a result, we utilized anti-CXCL13 antibodies to neutralize CXCL13 function in the conditioned medium and co-culture supernatant. We found that anti-CXCL13 antibodies can decrease M2 macrophages proliferation, migration, and invasiveness to ccRCC cells (Fig. 4A–C). Furthermore, we discovered that adding the chemokine CXCL13 to ccRCC cells could induce a substantial reduction in E-cadherin expression while increasing N-cadherin and vimentin expression. CXCL13 neutralization significantly reversed the down-regulation of epithelial markers and the up-regulation of mesenchymal markers caused by ccRCC cells and M2 macrophages co-culture (Fig. 4D).

### M2 macrophages secreted CXCL13 to promotes EMT of ccRCC cells via Akt signaling pathway

**Fig. 5:**
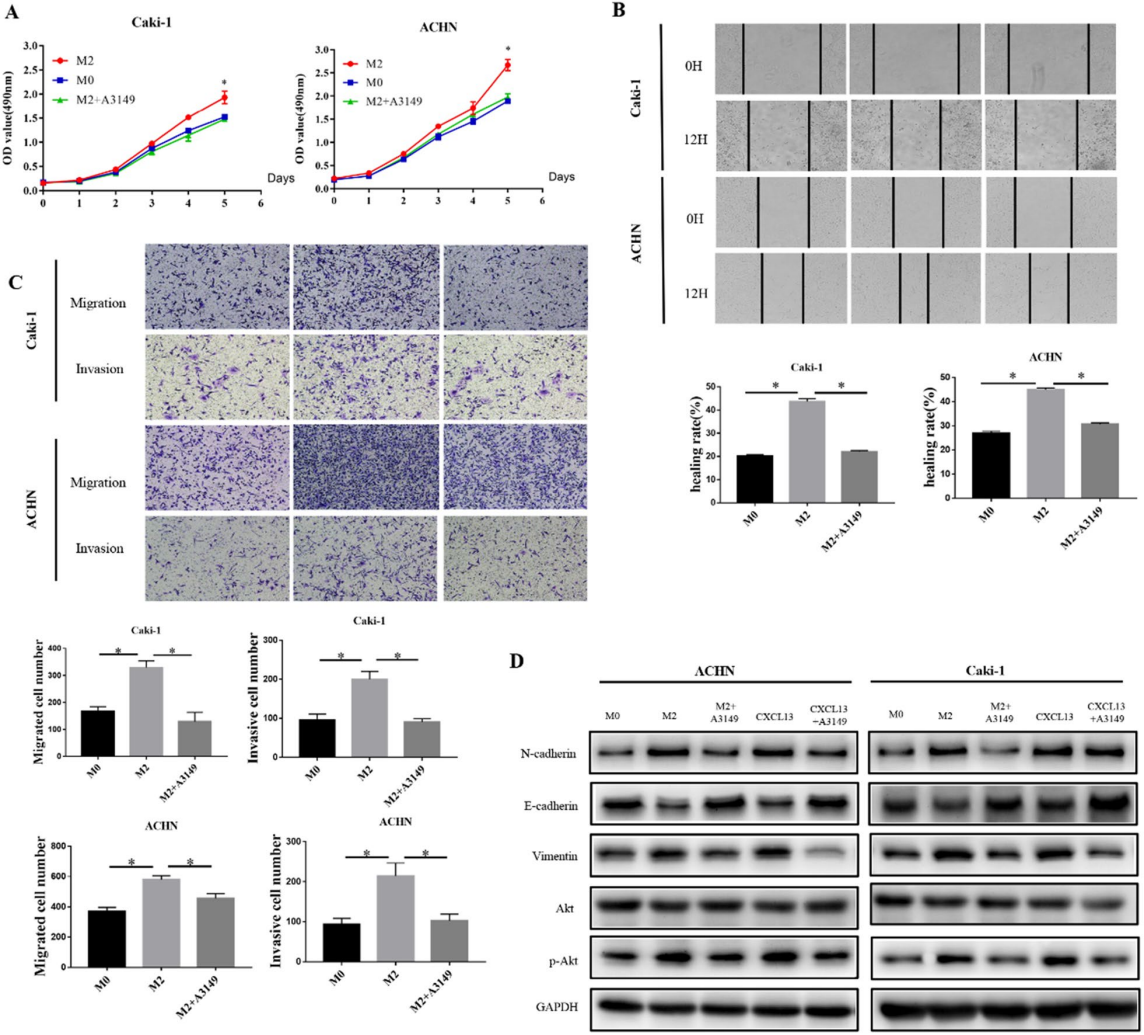
M2 macrophage secretion of CXCL13 promotes EMT in ccRCC cells via the Akt signaling pathway. **A** In the presence or absence of Akt inhibitors, MTS detects the effect of M2 macrophages on the proliferation of ACHN and Caki-1. **B** In the presence or absence of Akt inhibitors, the scratch healing test detects the effect of M2 macrophages on the migration of ACHN and Caki-1. **C** In the presence or absence of Akt inhibitors, the Transwell chamber test detects the effect of M2 macrophages on the migration and invasion of ACHN and Caki-1. **D** In the presence or absence of Akt inhibitors, western blotting revealed the effects of M2 macrophages and CXCL13 on ACHN and Caki-1EMT (*P < 0.05)

Previous research has shown that CXCL13 can enhance the proliferation of ccRCC cells via the PI3K-Akt pathway [11]. We utilized a specific AKT inhibitor (A3149) in this work and discovered that A3149 can inhibit the stimulation of M2 macrophages on the proliferation, migration, and invasion of ccRCC cells (Fig. 5A–C). A3149 also inhibited the expression of phosphorylated Akt (p-Akt) and EMT in ccRCC cells co-cultured with M2 macrophages or treated with CXCL13 (Fig. 5D). These results indicate that CXCL13 produced by M2 macrophages may enhance ccRCC cell proliferation, migration, invasion, and EMT via Akt signaling pathway.

### CXCL13 receptor CXCR5 is a poor prognostic factor for ccRCC

**Fig. 6:**
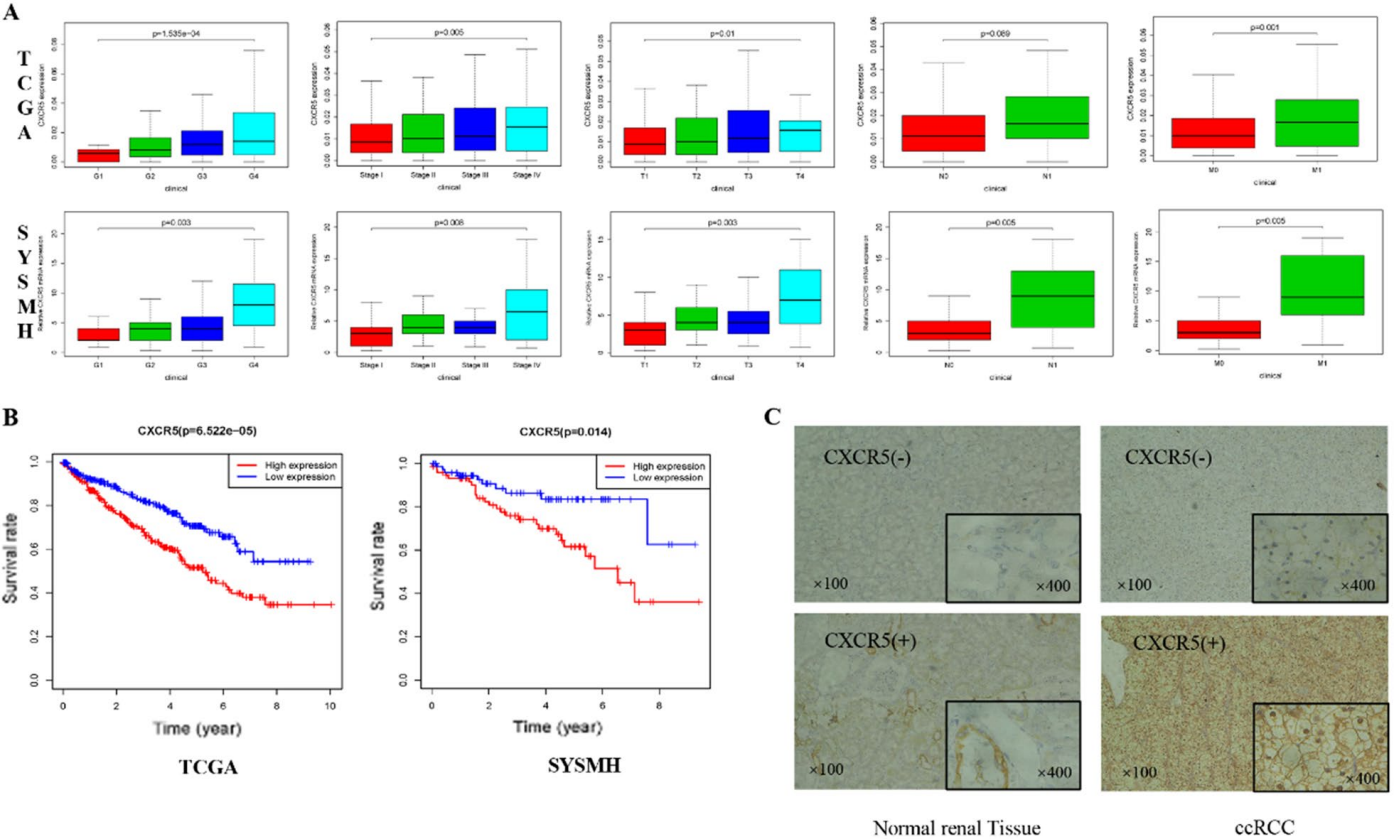
qPCR and Immunohistochemical to detect the expression of CXCR5 in ccRCC tissues. **A** TCGA database and our hospital database detect the relationship between CXCR5mRNA expression and ccRCC clinicopathological characteristics. **B** TCGA database and our hospital database to detect the relationship between CXCR5mRNA expression and ccRCC prognosis. **C** Immunohistochemical staining of CXCR5 protein in ccRCC tissues and adjacent tissues

CXCR5 is the sole CXCL13 receptor. We initially investigated the connection between CXCR5 mRNA expression and the clinicopathological characteristics and prognosis of ccRCC using our hospital’s (SYSMH) and TCGA databases. The findings revealed that increased CXCR5 expression was associated with a higher pathogenic grade and stage. The patients with high CXCR5 expression had a substantially worse outcome than patients with low expression (Fig. 6A, B). IHC was then used to examine the expression of the CXCR5 protein in RCC tissues. The findings revealed that there was a substantial difference in CXCR5 staining between cancer and surrounding normal tissues (Fig. 6C). In 90 ccRCC tissues, there are substantial variations in the clinicopathological features of CXCR5 positive and negative patients (Table 2). These clinical findings on CXCR5 demonstrate that the prognosis of ccRCC patients is poor, which is consistent with the in vitro results and supports the critical function of the CXCL13–CXCR5 axis in the development of ccRCC.

**Table 2:** Correlation between CXCR5 expression and clinical characteristics of ccRCC patients

Characteristics	Number	CXCR5		*P*-value
		Positive	Negative	
Gender				
Male	55	27	28	0.829
Female	35	18	17	
Age				
≤ 60	60	29	31	0.989
> 60	30	16	14	
Grade				
G1–2	56	23	33	0.030*
G3–4	34	22	12	
T stage				
T1–2	80	37	43	0.044*
T3–4	10	8	2	

*Results were considered statistically significant at *P *< 0.05

### CXCL13 promotes the growth of ccRCC in vivo

**Fig. 7:**
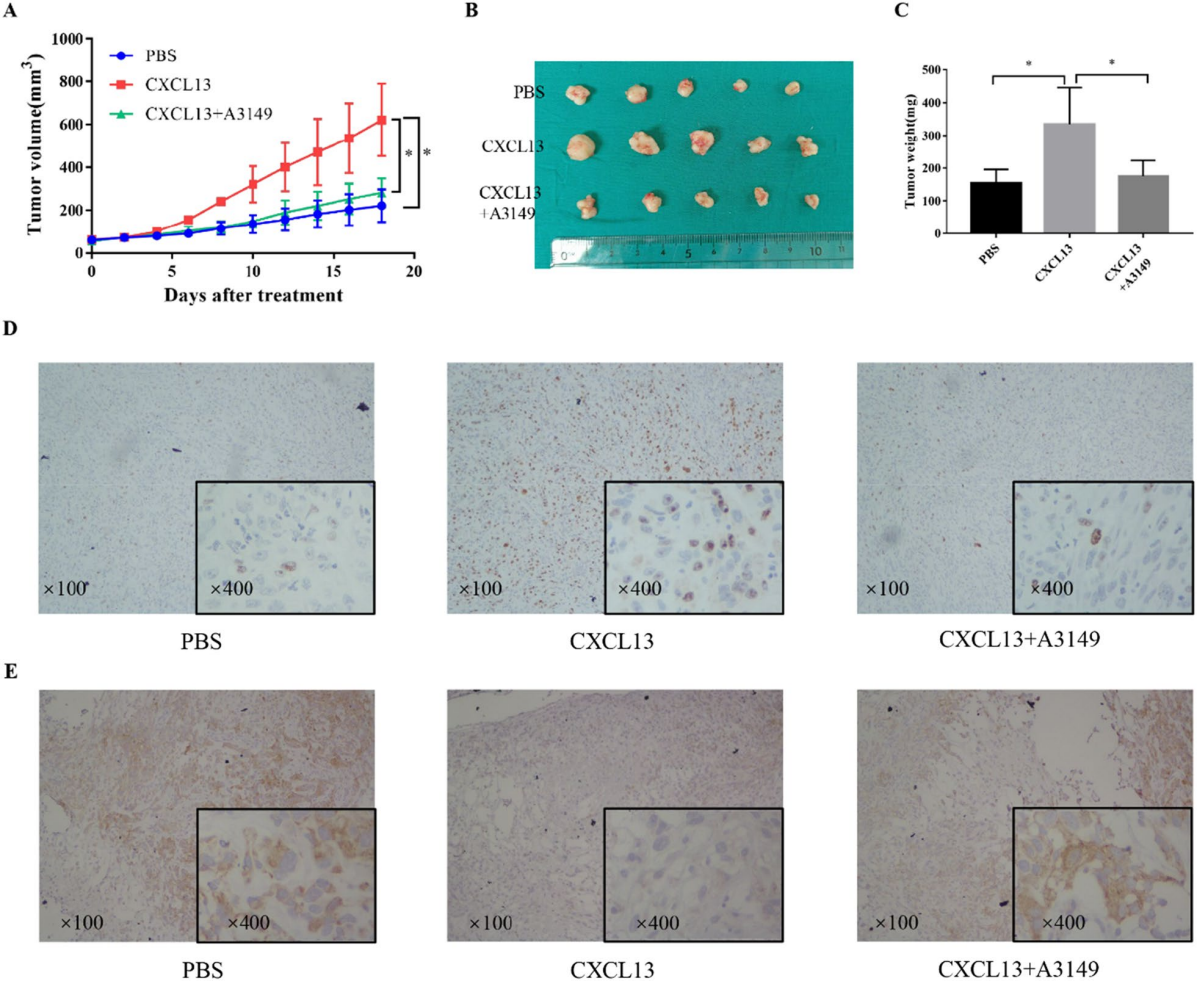
The effect of CXCL13 on tumor proliferation in vivo. **A** Tumor size was measured throughout animal experiments for calculating the tumor volume and drawing the tumor growth curve. **B** The mice were sacrificed and the xenograft tumors were removed and photographed. **C** Weight of xenografts was measured, recorded, and used for comparison among the PBS, CXCL13, and CXCL13 + A3149 groups. **D** The expression of Ki67 was detected by IHC in xenografts obtained from the PBS, CXCL13, and CXCL13 + A3149 groups, respectively. **E** The expression of E-cadherin was detected by IHC in xenografts obtained from the PBS, CXCL13, and CXCL13 + A3149 groups, respectively (*P < 0.05)

Caki-1 cells were placed under the skin of nude mice to detect the tumor-promoting effect of the chemokine CXCL13 in vivo. In this experiment, we also added the AKT inhibitor A3149 to further evaluate the mechanism of CXCL13 in promoting tumor proliferation and EMT. The tumor growth curve was drawn based on the tumor size measurement during the animal experiment, which indicated that the chemokine CXCL13 could promote tumor growth and that A3149 could reverse the promotion of CXCL13 on tumor growth (Fig. 7A). Then, the tumor was peeled off from the mice and photographed and weighed (Fig. 7B, C). Finally, IHC analysis was performed to detect the Ki67 and E-cadherin expression, which revealed that CXCL13 promoted the tumor Ki67 expression and reduced E-cadherin expression, while A3149 inhibited CXCL13’s promotion of tumor Ki67 expression and the inhibition of E-cadherin expression (Fig. 7D, E). These findings indicate that CXCL13 could significantly enhance the proliferation and EMT of ccRCC in vivo through the AKT pathway, which is consistent with our in vitro findings.

## Discussion

Macrophages are immune cells that are part of the human immune system’s humoral immunity and play a key role in autoimmunity, inflammatory response, and tumor immunity [12]. Macrophages are a varied collection of cells with high plasticity and heterogeneity. Most macrophages are classified into two phenotypes based on their activation status. M1 type and replacement activated macrophages (M2 type) are two types of classically activated macrophages [13]. Tumor-associated macrophages are macrophages that infiltrate TME (TAMs). TAMs are the most predominant infiltrating leukocyte group in most advanced solid tumors. TAM is now well acknowledged to primarily exhibit the kind of M2 macrophages that promote tumor development [13–15]. We co-cultured macrophages with tumor cells in this study to obtain M2 macrophages. The findings demonstrate that induced differentiation of M2 macrophages can promote the proliferation, migration, and metastasis of renal cancer cell lines. And, using qPCR and ELISA, we demonstrated that M2 macrophages play a key role in the chemokine CXCL13. CXCR5 is the sole CXCL13 receptor. CXCR5 was discovered to be a poor predictive factor for ccRCC in this research.

According to recent research, significant TAMs infiltration is related to a poor prognosis of different malignancies [16]. TAMs have an impact on nearly every aspect of tumor biology. It has the potential to induce angiogenesis, tumor progression, invasion, and metastasis. This influence on tumor development at various stages demonstrates their functional diversity. M2 macrophages have been reported to enhance the invasion and metastasis of gallbladder cancer [8], breast cancer [9], and head and neck squamous cell carcinoma [17] via the chemokine CCL18. M2 macrophages have also been reported to stimulate tumor angiogenesis via CCL-2 and CXCL8, as well as destroy the surrounding tissue matrix by producing tissue enzymes, therefore establishing a prerequisite regulation for cancer cell metastasis [18, 19]. TAM plays a complex function in tumor immunosuppression. TAM generates many immunosuppressive factors and chemokines, including IL-6, IL-10, TNF-α, TGF-β, etc., which decrease antigen presentation and impair T cell activity, allowing tumor cells to elude the body’s immunological surveillance [20]. We observed that M2 macrophages may enhance the proliferation, migration, invasion, and EMT of ccRCC through CXCL13 in our study. This is consistent with the finding M2 macrophage infiltration is a poor prognostic factor for RCC in the majority of studies [21].

The CXCL13/CXCR5 axis has been investigated in several tumors, although the associated consequence varies. According to research, the CXCL13/CXCR5 axis promotes colon cancer incidence, progression, and metastasis by secreting MMP13 and activating the PI3K/AKT pathway [22]. It can also suppress tumor immunity via the STAT3 signaling pathway, increasing colon cancer development and metastasis [23]. According to research, CXCL13 is an androgen-responsive gene. CAFs release CXCL13 in prostate cancer and have a role in androgen-independent prostate cancer development [24]. CXCL13, on the other hand, has been shown in certain studies to prevent the occurrence and growth of malignant tumors. Upregulation of CXCL13 expression in estrogen receptor-negative and human epidermal growth factor-positive breast tumors predicts a favorable prognosis. The Tfh cell subset capable of secreting CXCL13 converts Treg-mediated immunosuppression into adaptive anti-tumor immune activity. The essential component and has the potential to play a long-term function in preventing the development of breast tumor cells [25–27]. CXCL13 research in RCC is mostly aimed at promoting tumor growth and evading immunity. CXCL13+CD8+ T cell infiltration in tumors has been linked to poor clinical outcomes in ccRCC patients, according to research. CXCL13+CD8+ T cell abundance is an independent prognostic factor and potential immunotherapy target for ccRCC treatment [28]. Previously, we discovered that elevated CXCL13 expression in serum is a poor prognostic marker for ccRCC. The addition of CXCL13 to ccRCC cell lines can increase cell line growth [11]. In this study, we found that M2 macrophages in the tumor tissue microenvironment produce high levels of CXCL13 and that by attaching to the CXCR5 receptor on tumor cells, they promote the proliferation, migration, invasion, and EMT. Furthermore, CXCR5 is the sole receptor for CXCL13. CXCR5 expression was shown to be strongly linked with high clinic pathological features in the TCGA database and data from our hospital. CXCR5 was found to be a poor predictive factor for renal cancer in a prognostic study.

The significance and mechanism of TAM in tumorigenesis and development have become evident as research has progressed. We anticipate that targeted TAM is a very promising research area in the field of malignant tumor treatment and that it will likely become a novel technique of treating malignancies. There are now three major TAM therapies: inhibiting macrophage recruitment, depleting TAM, and promoting TAM anti-tumor transformation. For example, by blocking the most common CCL-2 and CCR-2 signaling pathways, macrophages recruitment and infiltration can be decreased, and tumor development can be delayed [29]. Some mannose-modified nanoliposome carriers and PLGA nanoparticle carriers selectively target the highly expressed mannose receptor (CD206) in M2 macrophages, which can accurately destroy M2 macrophages while having a low unfavorable effect on the body [30, 31]. The experimental results of Li et al. [32] show that the activation of PTEN and NHERF-1 may impede the development of TME macrophages other than M1 to M2. Whether the targeted TAM treatment may be coupled with currently utilized radiation and chemotherapy, anti-tumor blood vessels, small molecule targeted drugs, immunotherapy, and other treatments is deserving of further investigation.

Several studies have now revealed the potential advantages of targeting the CXCL13/CXCR5 pathway in different malignancies. Although no small molecule inhibitors that can directly target CXCL13 or CXCR5 have been discovered in pharmacology. However, recent gene knockdown/suppression findings including the neutralization of overexpressed CXCL13 and/or CXCR5 activities have shown the therapeutic potential/validation of this pathway in many kinds of human cancers [22, 33−35]. At the moment, various novel approaches are being employed to inhibit CXCL13/CXCR5 signaling in the tumor microenvironment. Recently, it was revealed that drug-loaded nanoparticles had promising outcomes in cancer therapy [36]. Silica nanoparticles loaded with snake venom may cause apoptosis and limit proliferation in human prostate cancer cells [37], as well as dramatically lower the levels of numerous chemokines (including CXCL13) and their receptors (including CXCR5). Simultaneously, chemokine-mediated migration lowers the risk of prostate cancer and breast cancer [38].

## Conclusions

In conclusion, our findings suggest that M2 macrophages play a crucial role in the genesis and progression of ccRCC. M2 macrophages can enhance ccRCC cell proliferation, migration, invasion, and EMT via CXCL13 in an Akt-dependent manner. These findings contribute to our understanding of the immune micro environment’s function in ccRCC and may aid in the identification of possible novel therapeutic targets for ccRCC.

## Supplementary Information

**Additional file 1. **The primer sequences of the analyzed genes.

**Additional file 2.** The original western blots with markers.

**Additional file 3. **The flow cytometry gating strategies.

**Additional file 4. **The expression of chemokines in ccRCC tissues in the TCGA database.

## Data Availability

The datasets used and/or analyzed during the current study are available from the corresponding author on reasonable request.

## Abbreviations

RCC: Renal cell carcinoma
ccRCC: Clear cell renal cell carcinoma
TME: Tumor microenvironment
TAM: Tumor-associated macrophages
EMT: Epithelial–mesenchymal transition
TCGA: The Cancer Genome Atlas
RT-qPCR: Reverse-transcription quantitative PCR
WB: Western blotting
ELISA: Enzyme-linked immunosorbent assay
IHC: Immunohistochemistry
Akt: Protein kinase B
CM: Conditioned medium

## References

[1] Kabaria R, Klaassen Z, Terris MK (2016). Renal cell carcinoma: links and risks. Int J Nephrol Renovasc Dis.

[2] Sung H, Ferlay J, Siegel RL, Laversanne M, Soerjomataram I, Jemal A, Bray F (2021). Global cancer statistics 2020: GLOBOCAN estimates of incidence and mortality worldwide for 36 cancers in 185 countries. CA Cancer J Clin.

[3] Moch H, Cubilla AL, Humphrey PA, Reuter VE, Ulbright TM (2016). The 2016 WHO classification of tumours of the urinary system and male genital organs-part a: renal, penile, and testicular tumours. Eur Urol.

[4] Takezawa Y, Izumi K, Shimura Y, Aerken M, Natsagdorji A, Iijima M (2016). Treatment outcome of low-dose interleukin-2 therapy in patients with metastatic renal cell carcinoma. Anticancer Res.

[5] Boutilier AJ, Elsawa SF (2021). Macrophage polarization states in the tumor microenvironment. Int J Mol Sci.

[6] Komohara Y, Hasita H, Ohnishi K, Fujiwara Y, Suzu S, Eto M (2011). Macrophage infiltration and its prognostic relevance in clear cell renal cell carcinoma. Cancer Sci.

[7] Zhou J, Tang Z, Gao S, Li C, Feng Y, Zhou X (2020). Tumor-associated macrophages: recent insights and therapies. Front Oncol.

[8] Zhou Z, Peng Y, Wu X, Meng S, Yu W, Zhao J (2019). CCL18 secreted from M2 macrophages promotes migration and invasion via the PI3K/Akt pathway in gallbladder cancer. Cell Oncol.

[9] Chen J, Yao Y, Gong C, Yu F, Su S, Chen J (2011). CCL18 from tumor-associated macrophages promotes breast cancer metastasis via PITPNM3. Cancer Cell.

[10] Hussain M, Adah D, Tariq M, Lu Y, Zhang J, Liu J (2019). CXCL13/CXCR5 signaling axis in cancer. Life Sci.

[11] Zheng Z, Cai Y, Chen H, Chen Z, Zhu D, Zhong Q (2019). CXCL13/CXCR5 axis predicts poor prognosis and promotes progression through PI3K/AKT/mTOR pathway in clear cell renal cell carcinoma. Front Oncol.

[12] Murray PJ (2017). Macrophage polarization. Annu Rev Physiol.

[13] Vankerckhoven A, Wouters R, Mathivet T, Ceusters J, Baert T, Van Hoylandt A (2020). Opposite macrophage polarization in different subsets of ovarian cancer: observation from a Pilot study. Cells.

[14] Munir MT, Kay MK, Kang MH, Rahman MM, Al-Harrasi A, Choudhury M (2021). Tumor-associated macrophages as multifaceted regulators of breast tumor growth. Int J Mol Sci.

[15] Tan Y, Wang M, Zhang Y, Ge S, Zhong F, Xia G (2021). Tumor-associated macrophages: a potential target for cancer therapy. Front Oncol.

[16] Mantovani A, Marchesi F, Malesci A, Laghi L, Allavena P (2017). Tumour-associated macrophages as treatment targets in oncology. Nat Rev Clin Oncol.

[17] Qin Y, Wang J, Zhu G, Li G, Tan H, Chen C (2019). CCL18 promotes the metastasis of squamous cell carcinoma of the head and neck through MTDH-NF-κB signalling pathway. J Cell Mol Med.

[18] Corliss BA, Azimi MS, Munson JM, Peirce SM, Murfee WL (2016). Macrophages: an inflammatory link between angiogenesis and lymphangiogenesis. Microcirculation.

[19] Riabov V, Gudima A, Wang N, Mickley A, Orekhov A, Kzhyshkowska J (2014). Role of tumor associated macrophages in tumor angiogenesis and lymphangiogenesis. Front Physiol.

[20] Cannarile MA, Weisser M, Jacob W, Jegg AM, Ries CH, Rüttinger D (2017). Colony-stimulating factor 1 receptor (CSF1R) inhibitors in cancer therapy. J Immunother Cancer.

[21] Shen H, Liu J, Chen S, Ma X, Ying Y, Li J (2021). Prognostic value of tumor-associated macrophages in clear cell renal cell carcinoma: a systematic review and meta-analysis. Front Oncol.

[22] Zhu Z, Zhang X, Guo H, Fu L, Pan G, Sun Y (2015). CXCL13–CXCR5 axis promotes the growth and invasion of colon cancer cells via PI3K/AKT pathway. Mol Cell Biochem.

[23] Chen X, Takemoto Y, Deng H, Middelhoff M, Friedman RA, Chu TH (2017). Histidine decarboxylase (HDC)-expressing granulocytic myeloid cells induce and recruit Foxp3+ regulatory T cells in murine colon cancer. Oncoimmunology.

[24] Ammirante M, Shalapour S, Kang Y, Jamieson CA, Karin M (2014). Tissue injury and hypoxia promote malignant progression of prostate cancer by inducing CXCL13 expression in tumor myofibroblasts. Proc Natl Acad Sci USA.

[25] Gu-Trantien C, Migliori E, Buisseret L, de Wind A, Brohée S, Garaud S (2017). CXCL13-producing TFH cells link immune suppression and adaptive memory in human breast cancer. JCI Insight.

[26] Gu-Trantien C, Loi S, Garaud S, Equeter C, Libin M, de Wind A (2013). CD4+ follicular helper T cell infiltration predicts breast cancer survival. J Clin Invest.

[27] Heimes AS, Madjar K, Edlund K, Battista MJ, Almstedt K, Elger T (2017). Subtype-specific prognostic impact of different immune signatures in node-negative breast cancer. Breast Cancer Res Treat.

[28] Dai S, Zeng H, Liu Z, Jin K, Jiang W, Wang Z (2021). Intratumoral CXCL13+ CD8+ T cell infiltration determines poor clinical outcomes and immunoevasive contexture in patients with clear cell renal cell carcinoma. J Immunother Cancer.

[29] Dong D, Zhang G, Yang J, Zhao B, Wang S, Wang L (2019). The role of iron metabolism in cancer therapy focusing on tumor-associated macrophages. J Cell Physiol.

[30] Ovais M, Guo M, Chen C (2019). Tailoring nanomaterials for targeting tumor-associated macrophages. Adv Mater.

[31] Corradetti B, Pisano S, Conlan RS, Ferrari M (2019). Nanotechnology and Immunotherapy in ovarian cancer: tracing new landscapes. J Pharmacol Exp Ther.

[32] Li N, Qin J, Lan L, Zhang H, Liu F, Wu Z (2015). PTEN inhibits macrophage polarization from M1 to M2 through CCL2 and VEGF-A reduction and NHERF-1 synergism. Cancer Biol Ther.

[33] Wang GZ, Cheng X, Zhou B, Wen ZS, Huang YC, Chen HB (2015). The chemokine CXCL13 in lung cancers associated with environmental polycyclic aromatic hydrocarbons pollution. Elife.

[34] Garg R, Blando JM, Perez CJ, Abba MC, Benavides F, Kazanietz MG (2017). Protein kinase C epsilon cooperates with PTEN loss for prostate tumorigenesis through the CXCL13–CXCR5 pathway. Cell Rep.

[35] Wei Y, Lin C, Li H, Xu Z, Wang J, Li R (2018). CXCL13 expression is prognostic and predictive for postoperative adjuvant chemotherapy benefit in patients with gastric cancer. Cancer Immunol Immunother.

[36] Li B, Li Q, Mo J, Dai H (2017). Drug-loaded polymeric nanoparticles for cancer stem cell targeting. Front Pharmacol.

[37] Badr G, Al-Sadoon MK, Rabah DM, Sayed D (2013). Snake (*Walterinnesia aegyptia*) venom-loaded silica nanoparticles induce apoptosis and growth arrest in human prostate cancer cells. Apoptosis.

[38] Badr G, Al-Sadoon MK, Rabah DM (2013). Therapeutic efficacy and molecular mechanisms of snake (*Walterinnesia aegyptia*) venom-loaded silica nanoparticles in the treatment of breast cancer- and prostate cancer-bearing experimental mouse models. Free Radic Biol Med.

