# Supplementary material for: M2 macrophages secrete CXCL13 to promote renal cell carcinoma migration, invasion, and EMT
Source: Cancer Cell Int. 2021 Dec 18;21:677. doi: 10.1186/s12935-021-02381-1 (PMC8684162; doi:10.1186/s12935-021-02381-1)
Supplement: Supplementary file 2 — Additional file 2. The original western blots with markers. [file 12935_2021_2381_MOESM2_ESM.pptx]

## Slide 1
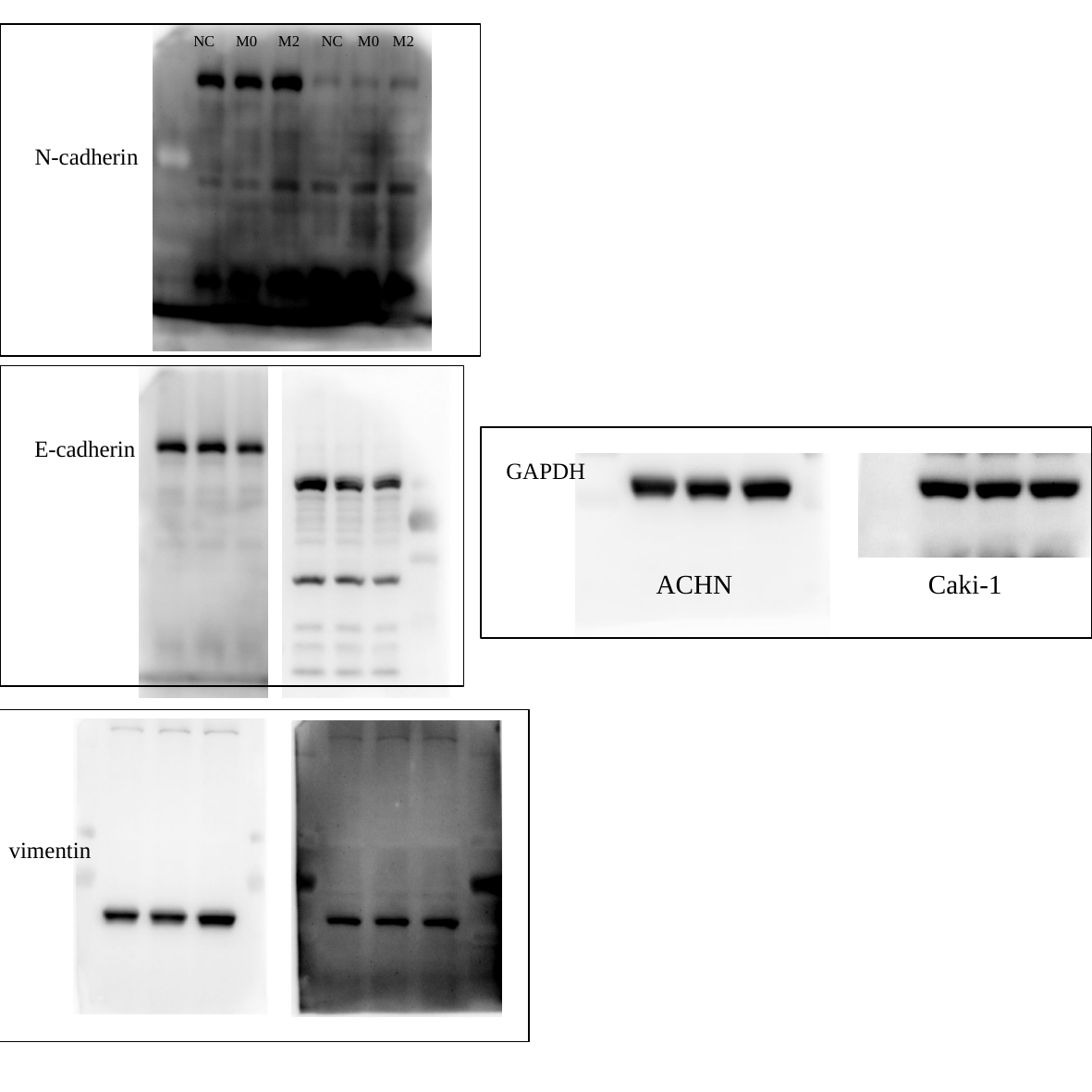

NC
M0
M0
M2
NC
M2
N-cadherin
E-cadherin
GAPDH
ACHN
Caki-1
vimentin

## Slide 2
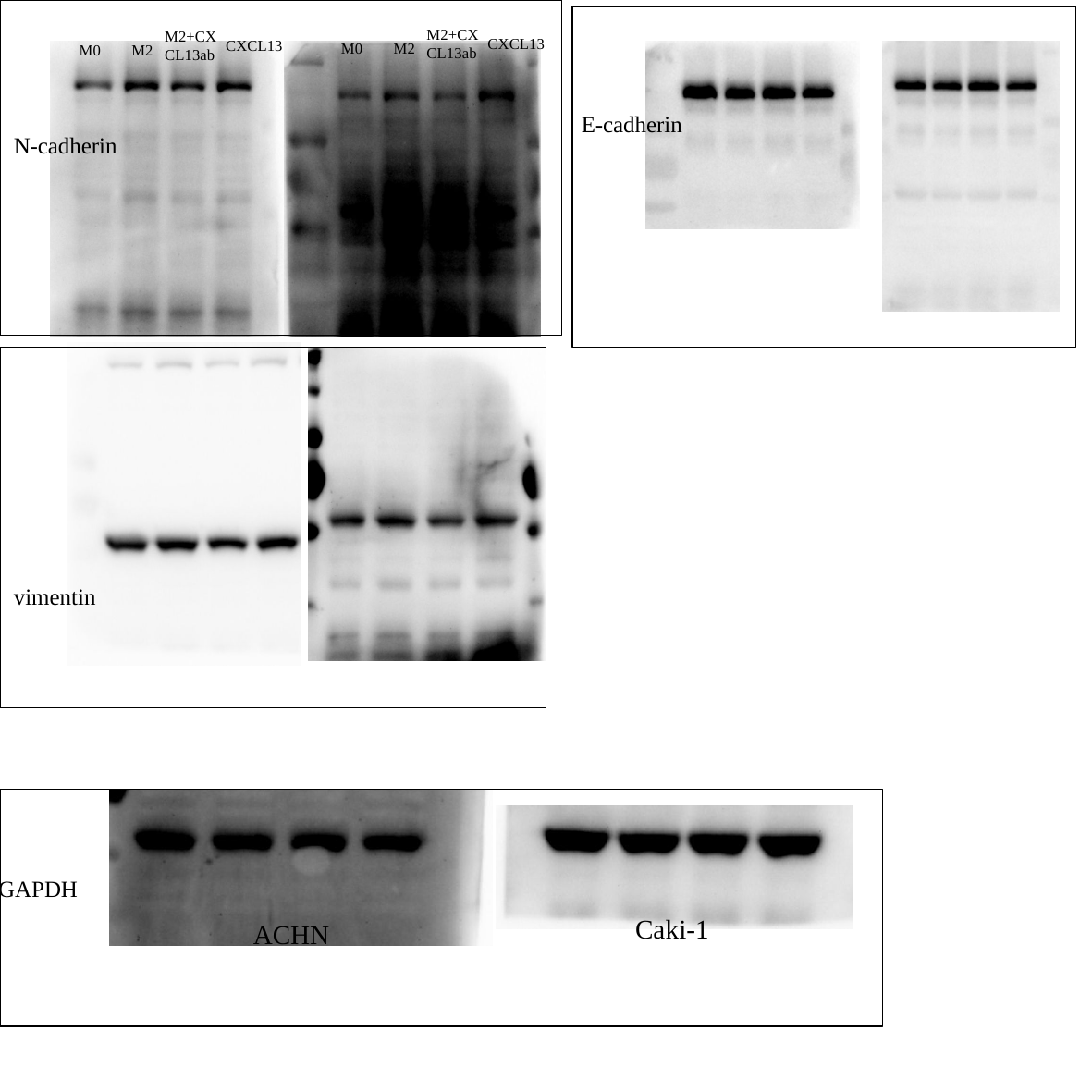

M2+CXCL13ab
M2+CXCL13ab
CXCL13
CXCL13
M0
M2
M0
M2
E-cadherin
N-cadherin
vimentin
GAPDH
Caki-1
ACHN

## Slide 3
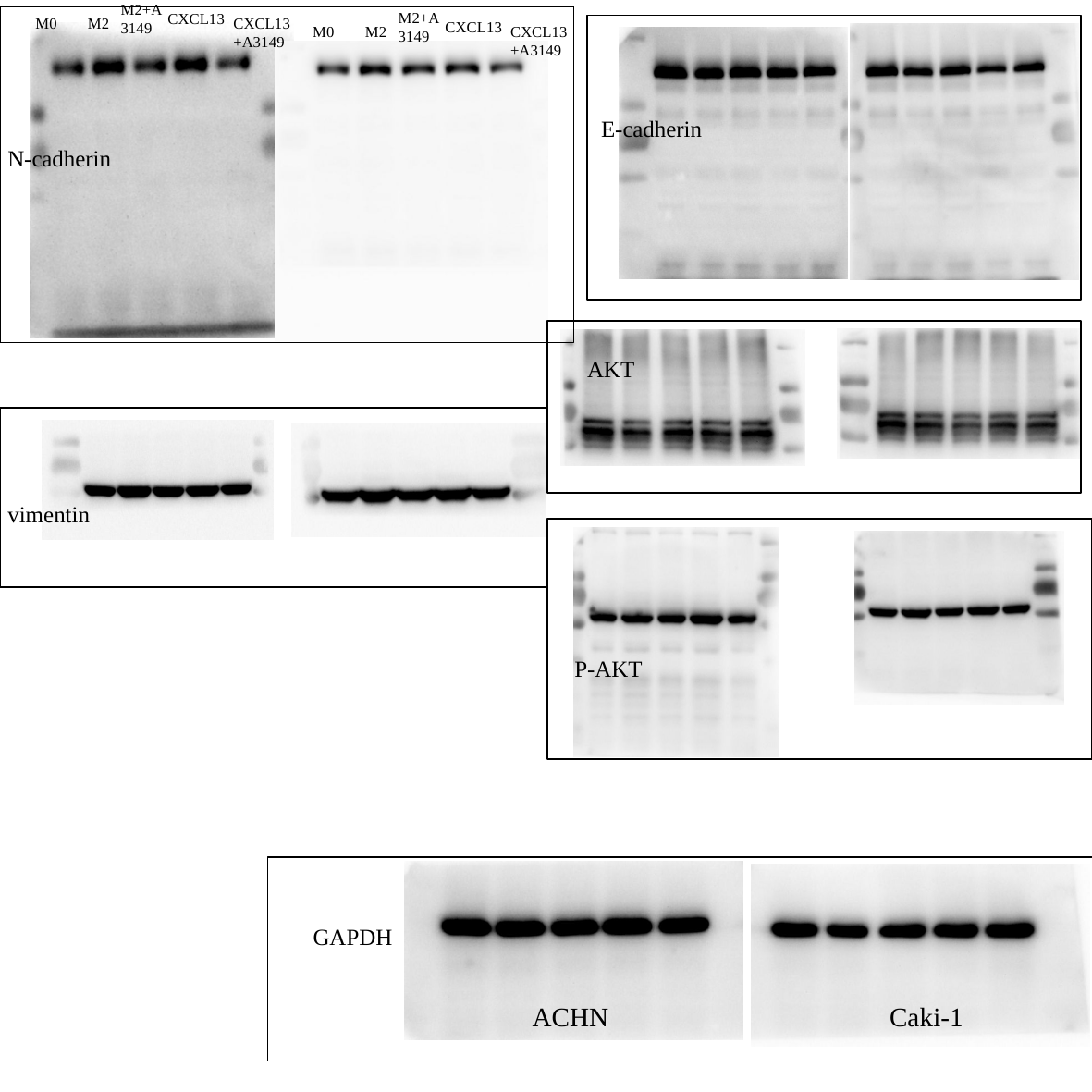

M2+A3149
M2+A3149
CXCL13
M0
M2
CXCL13+A3149
CXCL13
M0
M2
CXCL13+A3149
E-cadherin
N-cadherin
AKT
vimentin
P-AKT
GAPDH
Caki-1
ACHN
