# Supplementary figures and images for: M2 macrophages secrete CXCL13 to promote renal cell carcinoma migration, invasion, and EMT
Source: Cancer Cell Int. 2021 Dec 18;21:677. doi: 10.1186/s12935-021-02381-1 (PMC8684162; doi:10.1186/s12935-021-02381-1)

Blank control

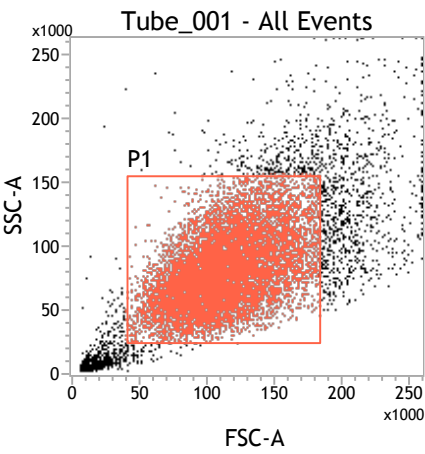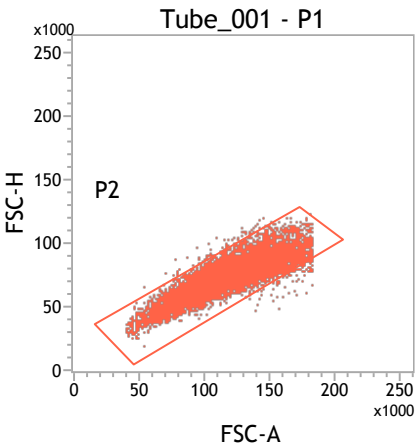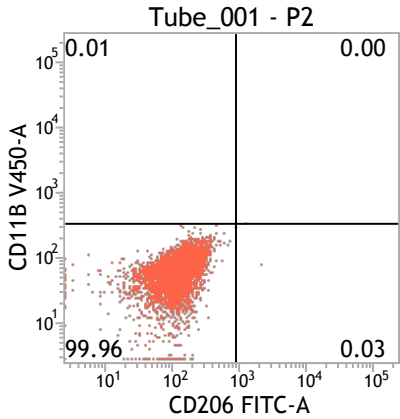

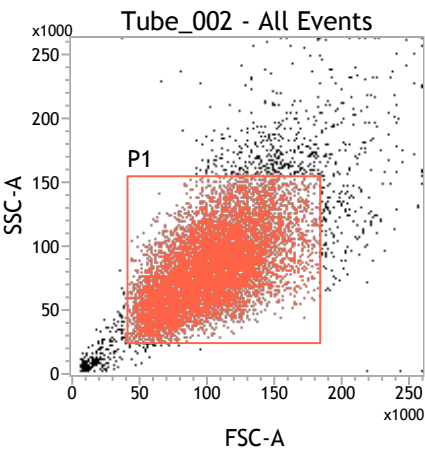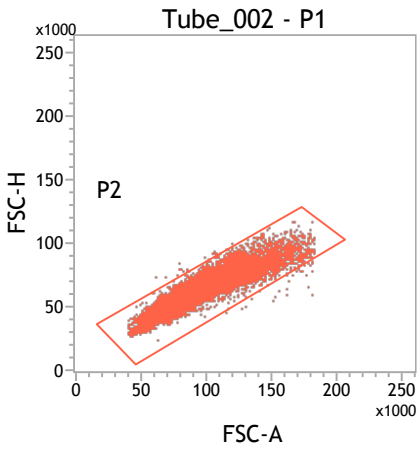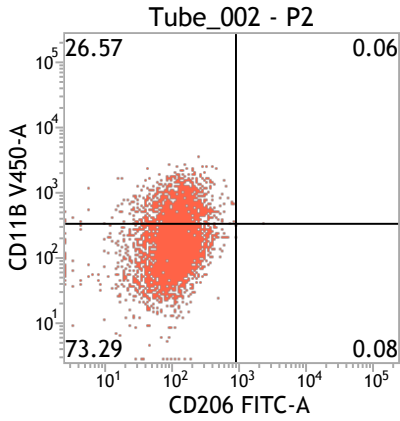

M2-Caki-1

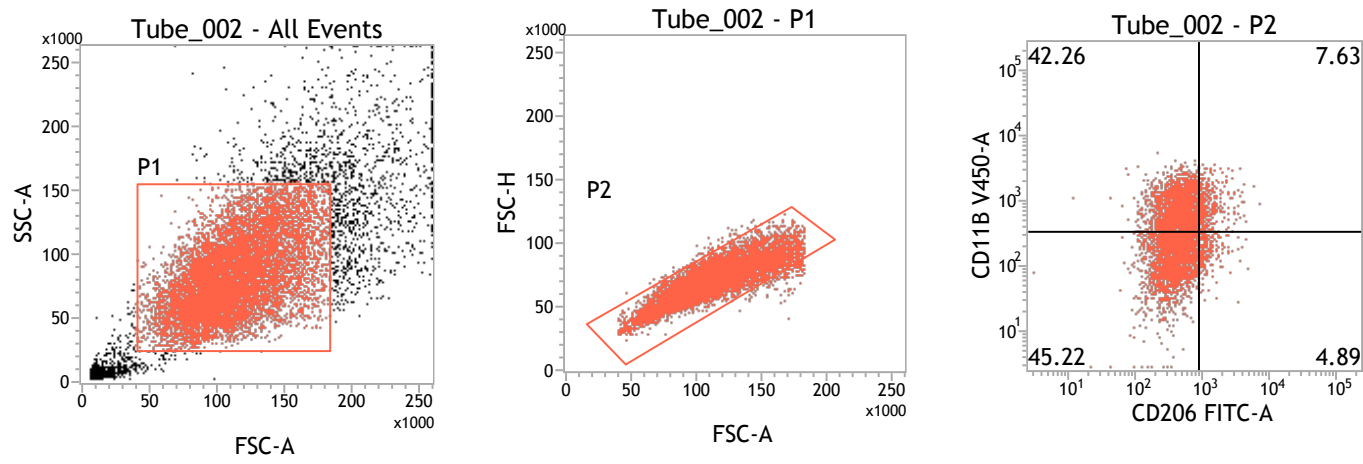

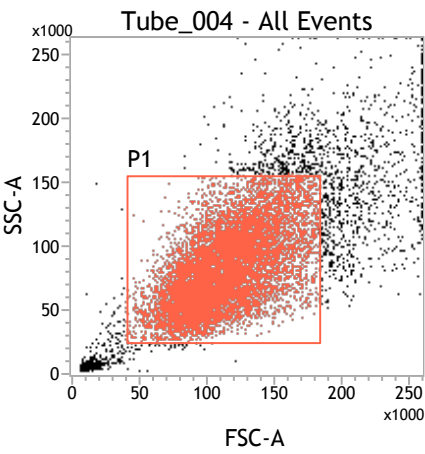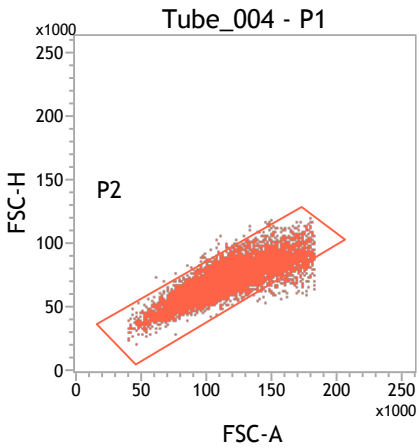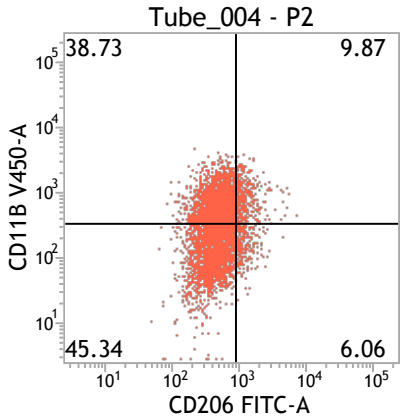

Supplement: Supplementary file 3 — Additional file 3. The flow cytometry gating strategies. [file 12935_2021_2381_MOESM3_ESM.pdf]
